# Ultrasound shear wave speeds reduced following hamstring strain injury but not after returning to sport

**DOI:** 10.1186/s13244-023-01571-x

**Published:** 2024-01-08

**Authors:** Scott K. Crawford, Christa M. Wille, Mikel R. Joachim, Kenneth S. Lee, Bryan C. Heiderscheit

**Affiliations:** 1 Department of Kinesiology, University of Wisconsin-Madison, 1300 University Ave, Madison, WI 53706 USA; 2 Department of Orthopedics & Rehabilitation, University of Wisconsin-Madison, 1685 Highland Ave, Madison, WI 53705 USA; 3 Department of Biomedical Engineering, University of Wisconsin-Madison, Madison, WI USA; 4 Badger Athletic Performance Program, University of Wisconsin-Madison, Madison, WI USA; 5 Department of Radiology, University of Wisconsin-Madison, Madison, WI USA

**Keywords:** College athletes, Elasticity, Hamstring Muscles, Ultrasonography

## Abstract

**Objectives:**

The purpose of the study was to investigate differences in ultrasound shear wave speed (SWS) between uninjured and injured limbs following hamstring strain injury (HSI) at time of injury (TOI), return to sport (RTS), and 12 weeks after RTS (12wks).

**Methods:**

This observational, prospective, cross-sectional design included male and female collegiate athletes who sustained an HSI. SWS imaging was performed at TOI, RTS, and 12wks with magnetic resonance imaging. SWS maps were acquired by a musculoskeletal-trained sonographer at the injury location of the injured limb and location-matched on the contralateral limb. The average SWS from three 5 mm diameter Q-boxes on each limb were used for analysis. A linear mixed effects model was performed to determine differences in SWS between limbs across the study time points.

**Results:**

SWS was lower in the injured limb compared to the contralateral limb at TOI (uninjured – injured limb difference: 0.23 [0.05, 0.41] m/s, *p* = 0.006). No between-limb differences in SWS were observed at RTS (0.15 [-0.05, 0.36] m/s, *p* = 0.23) or 12wks (-0.11 [-0.41, 0.18] m/s, *p* = 0.84).

**Conclusions:**

The SWS in the injured limb of collegiate athletes after HSI was lower compared to the uninjured limb at TOI but not at RTS or 12 weeks after RTS.

**Critical relevance statement:**

Hamstring strain injury with structural disruption can be detected by lower injured limb shear wave speed compared to the uninjured limb. Lack of between-limb differences at return to sport may demonstrate changes consistent with healing. Shear wave speed may complement traditional ultrasound or MRI for monitoring muscle injury.

**Key points:**

• Ultrasound shear wave speed can non-invasively measure tissue elasticity in muscle injury locations.

• Injured limb time of injury shear wave speeds were lower versus uninjured limb but not thereafter.

• Null return to sport shear wave speed differences may correspond to structural changes associated with healing.

• Shear wave speed may provide quantitative measures for monitoring muscle elasticity during recovery.

**Graphical Abstract:**

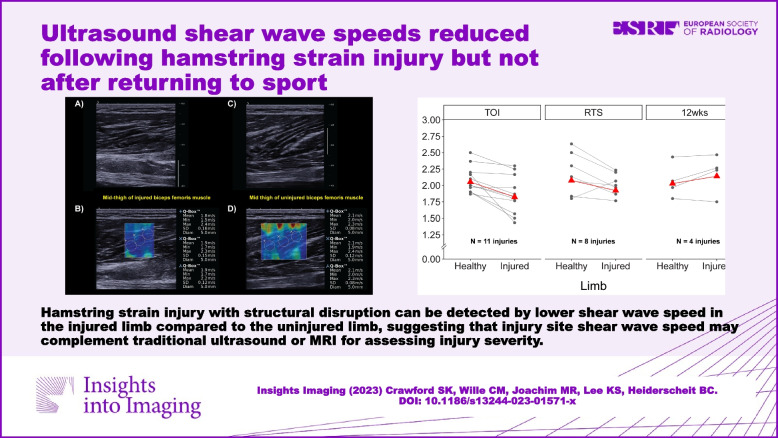

## Introduction

Hamstring strain injuries (HSI) are the most common injury in high-intensity, running-based sports [1–3]. Diagnosis is often corroborated with magnetic resonance imaging (MRI) and ultrasound imaging findings [4–9]. However, the usefulness of medical imaging for prognosis and the ability to determine the time to return to sport (RTS) and potential reinjury risk is questioned [10–12]. One possibility for the limited ability of medical imaging in providing additional value to clinical tests [13, 14] is that conventional MRI or ultrasound provide information only related to gross anatomy yet neglect inherent material properties of the muscle.

Muscle material properties are dependent upon the structure and composition of the tissue and play a significant role in the muscle’s response to lengthening strain and muscle performance [15, 16]. Though clinical tests (e.g., straight leg raise, active knee extension test) are often used to infer muscle mechanical properties such as stiffness, these tests may be suboptimal as the outcomes include contributions of both non-muscular tissues (e.g., tendon, ligaments, and the joint capsule) and the contributions of individual muscles which comprise an entire group (e.g., hamstrings) [17–19]. Considering previously injured hamstrings show greater displacements during hamstring-specific loading in the most susceptible areas for injury [20], methods that quantify the material properties in specific locations of injured muscle may provide insight into tissue loading response.

Ultrasound shear wave elastography is an imaging technique to non-invasively measure direct tissue elasticity in definite regions of the muscle and is not subject to influences of the whole muscle–tendon unit [17, 21]. The shear wave speed (SWS) serves as a proxy measure of skeletal muscle elasticity and increases with both passive lengthening and contraction intensity in the hamstrings [22, 23]. Shear modulus has also been related to rapid force development in healthy gastrocnemius muscles [24]. Shear wave elastography has also proven beneficial in musculoskeletal medicine by characterizing tendinopathy and plantar fasciitis with greater sensitivity than conventional sonographic measures of echotexture changes and vascularization detected by B-mode and power Doppler, respectively [25–27]. Specifically, degenerative changes in the tendon and fascia collagen matrix have consequent reductions in SWS, suggesting lower tissue stiffness [25–27]. However, less is known about how SWS is affected following a muscle strain injury in humans at the local injury site at the time of injury (TOI) and at RTS.

Therefore, the purpose of the study was to investigate differences in SWS between injured muscle and the corresponding healthy muscle in the uninjured limb following HSI at TOI, RTS, and 12 weeks after RTS (12wks) in an observational, prospective, cross-sectional design in collegiate athletes. It was hypothesized that SWS would be lower in the injured limb compared to the uninjured limb at TOI, but no between-limb differences would be present at RTS and 12wks.

## Methods

### Study design and procedures

The data presented in this study were collected as a part of a larger observational, prospective investigation of collegiate athletes who sustained an HSI [28, 29]. The current investigation was a cross-sectional analysis of available SWS data collected as a secondary outcome of the overall study design. The Health Sciences Institutional Review Board at the University of Wisconsin-Madison approved all procedures (IRB ID: 2017–0152). Athletes provided informed consent prior to participation in all study procedures.

Male and female collegiate athletes who participated in either American football, soccer, basketball, or track and field and sustained an acute HSI were recruited to participate in the study. An HSI was defined as sudden onset of pain in the posterior thigh that occurred during sport participation, required medical attention, and resulted in the athlete losing one or more days from training or competition. HSIs were determined and confirmed by the athlete’s medical team. Criteria for HSI occurrence was based on the presence of any two or more symptoms including: palpable pain along any of the hamstring muscles, posterior thigh pain without radicular symptoms during straight leg raise testing, weakness with resisted knee flexion, pain with resisted knee flexion, and posterior thigh pain with sports/running [28].

MRI and ultrasound SWS imaging were obtained within 7 days of a respective timepoint (TOI, RTS, 12wks) [30]. Due to scheduling difficulties, the order of MRI or ultrasound imaging acquisition varied. A standardized rehabilitation protocol was implemented by the team’s athletic trainer, and RTS was defined as the date when medical clearance was obtained to resume all sport-related activities [31]. Criteria for RTS clearance included absence of hamstring-specific pain upon palpation, symmetry between limbs for manually resisted isometric strength and range of motion, and absence of pain or stiffness during high-speed running [31, 32].

### Inclusion criteria for analysis

**Fig. 1:**
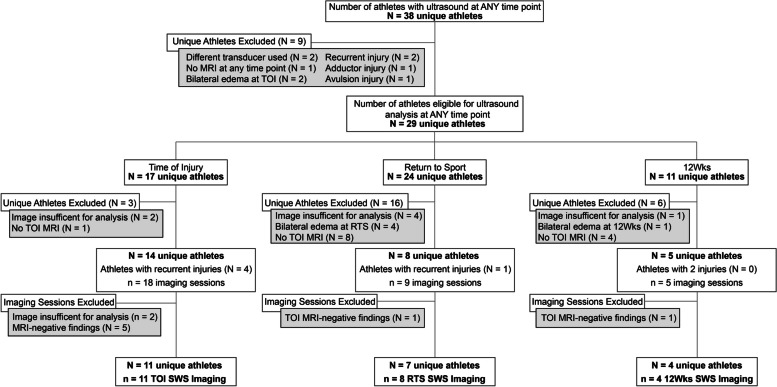
Flow chart for athletes included for analysis. Note that *N* refers to the number of unique athletes and *n* refers to number of imaging session. Abbreviations: 12wks, 12 weeks after return to sport clearance; MRI, magnetic resonance imaging; RTS, return to sport; SWS, shear wave speed; TOI, time of injury

If an athlete had a subsequent HSI that occurred at any point prior to the 12wks imaging session, then the index HSI was eligible for inclusion, but the subsequent HSI was not. If an athlete had a subsequent HSI after the 12wks imaging session, the subsequent HSI was eligible for analysis as a separate HSI. A flow chart of athletes included for analysis at each time point is shown in Fig. 1.

### Ultrasonic shear wave speed and magnetic resonance imaging procedures

Ultrasound SWS imaging was performed using a commercial ultrasound system (Aixplorer, Supersonic Imagine, Aix-en-Provence, France) with a linear array transducer (SL10-2, 38 mm aperture). A standardized image acquisition protocol was determined prior to data collection. Two musculoskeletal-trained sonographers (both with over 5 years of musculoskeletal ultrasound experience, trained, and supervised by the same musculoskeletal radiologist) were responsible for collecting images throughout the study. Both sonographers were informed of the study design, were provided with the same image acquisition protocol, and were trained by the same musculoskeletal radiologist with over 20 years’ experience (KSL). To maintain consistency across ultrasound image acquisitions and so as not to bias the sonographers, SWS were collected without the use of MRI findings. Although participants in this study completed both US and MRI imaging, MRI imaging was not always acquired before US imaging.

Athletes laid in a relaxed, prone position on a hospital bed with their hips and knees in a neutral position (0° flexion). The distance from the ischial tuberosity to the popliteal fossa was measured and the mid-belly location was recorded at 50% of the measured length. The proximal location was taken at 25% of the measured length from the ischial tuberosity. The injury site was defined as the location of maximal pain to palpation. The transducer was coated with a copious amount of acoustic gel, and a transverse view of each hamstring muscle at each location was obtained to ensure correct positioning for muscle visualization. The transducer was then rotated to acquire a longitudinal image of the muscle [28]. SWS were acquired in the longitudinal view with minimal pressure applied to the transducer when acquiring SWS. A single SWS map was generated in the middle of the image to maximize lateral resolution and adjusted so the depth of the SWS map spanned most of the muscle thickness. The SWS map depth was adjusted by the sonographers to minimize signal loss while maximizing the SWS map area within the middle thickness of the muscle. The SWS map was placed over the region of injury if it could be identified on the ultrasound images and was free of artifacts. Otherwise, it was placed as near the injury site to minimize artifacts while capturing tissue adjacent to the injured tissue location.

**Fig. 2:**
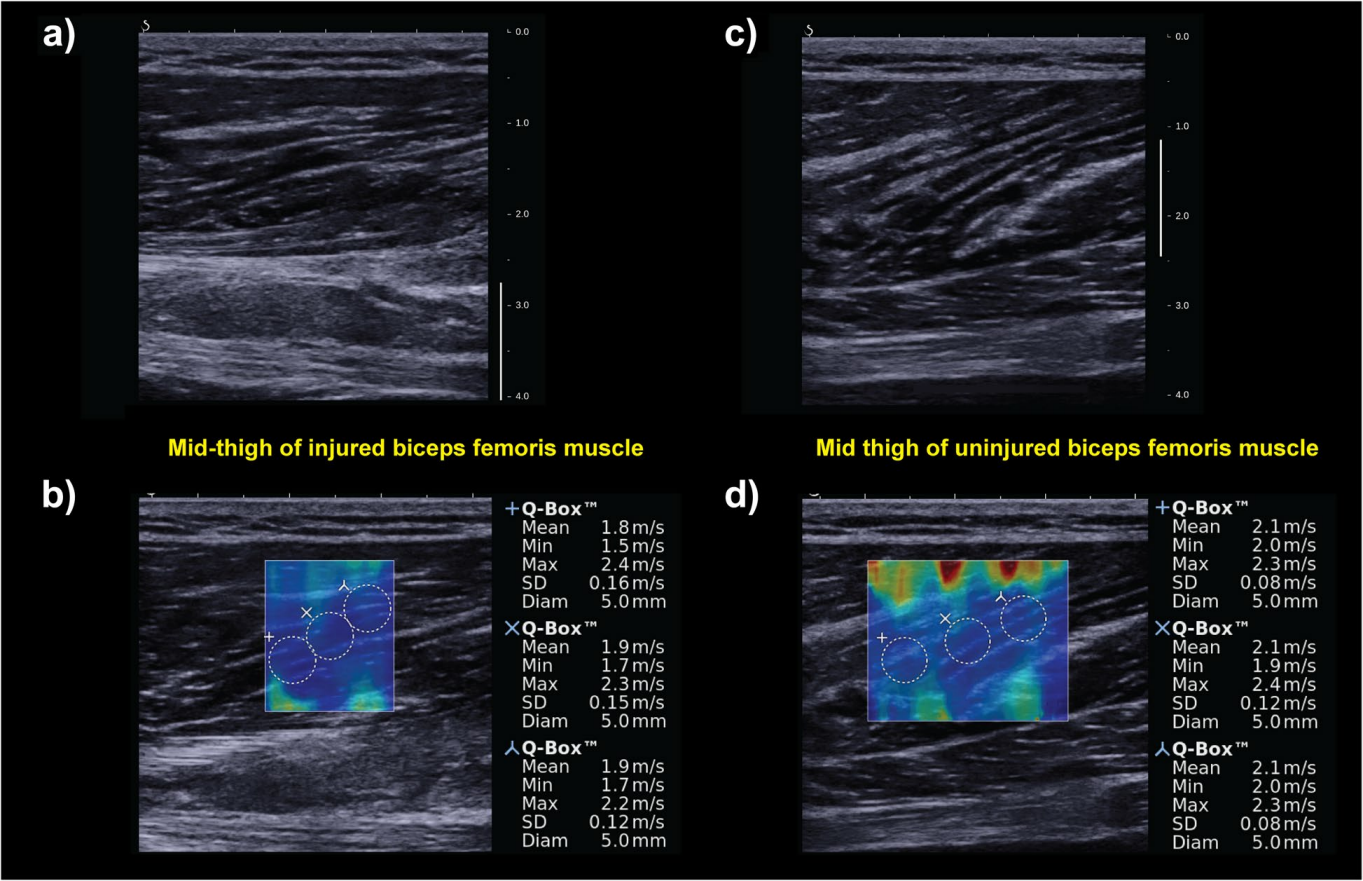
Representative B-mode and shear wave speed (SWS) imaging from one athlete at TOI. **a** B-mode image of the injury site. The injury location was defined as location of maximum tenderness to palpation and location-matched on the uninjured limb. **b** SWS with 3 Q-boxes of the injured limb. SWS were measured using 5 mm diameter Q-boxes positioned within the middle of the SWS map. **c** B-mode image of the uninjured limb at the injury-matched location. **d** SWS of the injury-matched site in the uninjured limb

SWS were measured in 3 separate 5 mm diameter Q-boxes placed within the SWS map (Fig. 2). Locations of the Q-boxes were placed by the sonographer based on their clinical expertise. The Q-box locations corresponded to regions with the SWS map without artifact and in regions representative of the injured tissue. The SWS of the 3 separate Q-boxes were averaged for subsequent analyses. This procedure was repeated for each muscle (biceps femoris long head, semitendinosus, and semimembranosus) and location across both limbs. The SWS for biceps femoris short head were not assessed due to the low incidence of injury and greater variability in SWS values [33]. The injury location was corroborated post hoc by TOI MRI and measured SWS were extracted from the primary muscle of injury at the injury location. The average of the SWS from the 3 Q-boxes on each limb were used for subsequent analysis.

Participants also received an MRI examination of both thighs. A 3.0T scanner (MR750 GE Healthcare Discovery, Waukesha, WI) was used with a 32-channel full torso coil. The athlete was positioned supine and feet first into the scanner. Axial and coronal views were acquired using a T1-weighted spoiled gradient recalled echo (SPGR) sequence (no fat saturation, TR/TE = 750/16 ms, 40 cm field of view, 480 × 480 matrix, 3 mm slice thickness) for assessment of anatomy. A fat-suppressed T2-weighted fast relaxation fast spin echo (FR-FSE) sequence (TR/TE = 4400/70 ms, 40 cm field of view, 512 × 512 matrix, 3 mm slice thickness) was used by a musculoskeletal radiologist (K.L.) with over 20 years of training to identify muscle edema (region of hyperintense signal) and to determine the primary hamstring muscle injured. In instances where multiple muscles had visible edema on T2-weighted MRI sequences, the primary muscle was defined as having the most visual edema. Injuries were graded using the British Athletics Muscle Injury Classification (BAMIC) system [5, 6].

### Statistical analyses

Athlete demographics are reported as mean (standard deviation) and days to RTS as median (interquartile range). A linear mixed effects model was performed to determine differences in SWS between limbs at the injury location across the three study time points. The linear mixed effects model accounts for individuals who may not have been included in multiple time points due to the cross-sectional nature of the study. The fixed effects were limb (injured or uninjured) and time point (TOI, RTS, and 12wks), and athlete was input as a random effect. A full factorial model was run and input into R statistical software as $SWS \sim Limb*Timepoint + (1 | Athlete)$ [34, 35]. Significant interactions and main effects were followed with pairwise post hoc Tukey tests. In the event of a significant interaction, only comparisons between limbs at the same time point were considered as these are clinically meaningful [36]. The least square means [95% confidence interval (CI)] were calculated for differences in SWS between limbs expressed as $Uninjured-Injured$.

## Results

Subject demographics are shown in Table 1. The median time between HSI and TOI SWS imaging was 4.0 (3.5–5.5) days. Median time to RTS 29.0 (18.8–49.3) days. A total of 16 athlete injuries from 15 unique athletes were included (one athlete had a subsequent HSI on the opposite limb). The biceps femoris long head was the most injured primary muscle across all unique injuries (14 of 16, 87.5%). One (6.3%) injury occurred in the semitendinosus muscle and one injury (6.3%) occurred in the semimembranosus muscle. Edema was present in 7 of the 8 (87.5%) RTS imaging sessions and 2 of the 4 (50%) imaging sessions at 12wks.

**Table 1:** Participant information for athletes included for ultrasound shear wave speed imaging

Characteristics of athletes included for shear wave speed imaging	
Number of unique athletes (total HSI)	15 (16)
Sex (female | male)	1 | 14
Sport (football | track and field | basketball | soccer)	6 | 9 | 0 | 0
Age (years)^a^	19.5 (1.3)
Height (m)^a^	1.83 (0.07)
Weight (kg)^a^	85.1 (18.3)
Body mass index (kg/m^2^)^a^	25.3 (4.0)
BAMIC classification of unique injuries (count)^b^	
Small myofascial tear (1a)	3
Small muscular/musculotendinous junction tear (1b)	1
Moderate myofascial tear (2a)	4
Moderate muscular/musculotendinous junction tear (2b)	0
Moderate-sized intratendinous tear (2c)	0
Extensive myofascial tear (3a)	0
Extensive muscular/musculotendinous tear (3b)	0
Extensive intratendinous tear (3c)	8

^a^Data are presented as mean (standard deviation)

^b^One athlete suffered a recurrent HSI resulting in 16 total unique injuries included

*Abbreviations*: *BAMIC* British Athletics Muscle Injury Classification

**Fig. 3:**
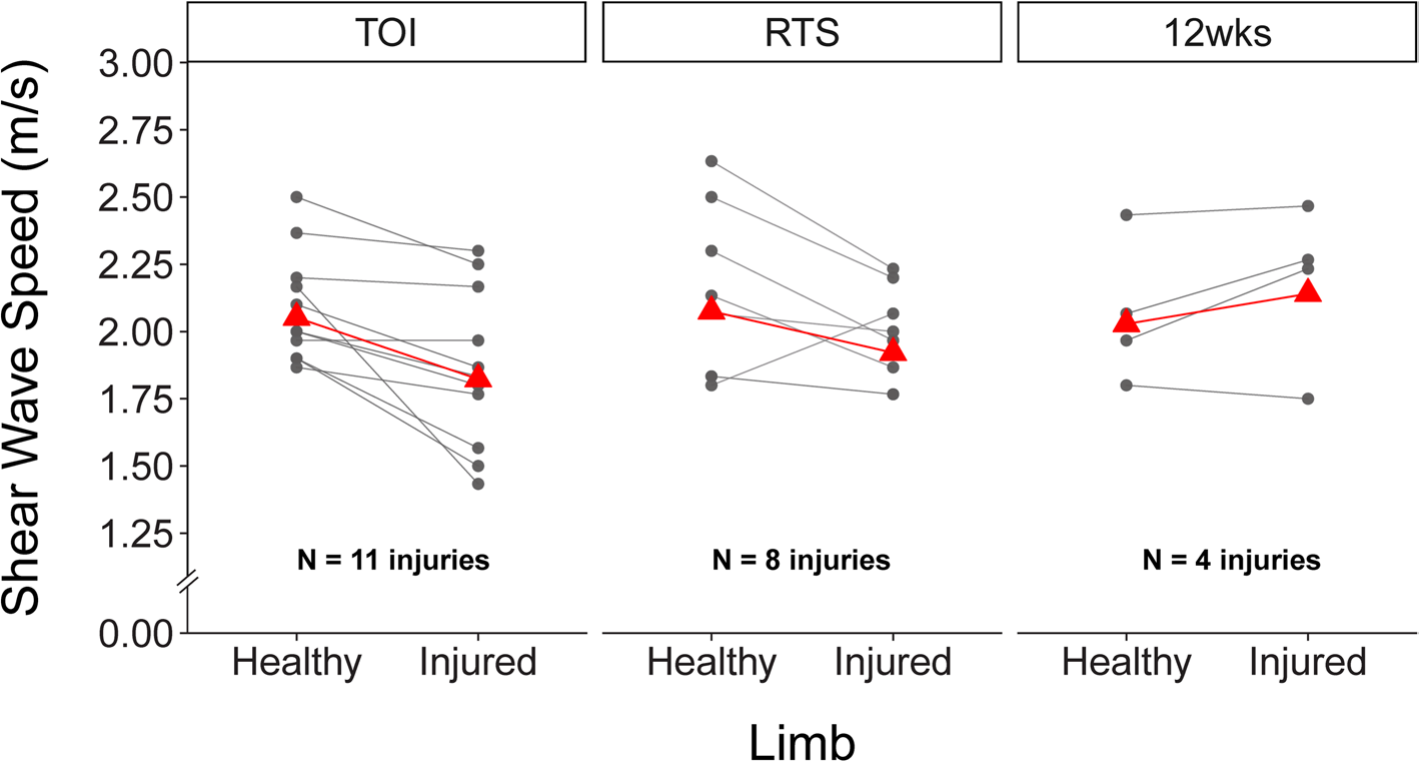
Between-limb shear wave speeds (SWS) for unique hamstring strain injury. Individual athlete SWS are plotted as circles. The least square means from the linear mixed effects model are plotted as red triangles. Significant between-limb differences in SWS were observed at time of injury (TOI), but not at return to sport (RTS) or 12 weeks after RTS (12wks)

Between-limb SWS for each unique HSI are shown in Fig. 3. A significant limb by time point interaction was detected for SWS (*p* = 0.02). Post hoc Tukey tests revealed a significant difference in SWS between limbs at TOI (mean difference: 0.23 [0.05, 0.41] m/s, *p* = 0.006) (Table 2). No differences in SWS were detected between limbs at RTS (0.15 [-0.05, 0.36] m/s, *p* = 0.23) or 12wks (-0.11 [-0.41, 0.18] m/s, *p* = 0.84).

**Table 2:** Least square means [95% confidence interval (CI)] of shear wave speed (SWS) estimates

Time point	Limb	Least square mean of SWS (m/s) [95% CI]	Between-limb difference^a^ in SWS (m/s) [95% CI]	*p*-value
TOI	Injured	1.82 [1.68, 1.97]	0.23 [0.05, 0.41]	0.006
	Uninjured	2.05 [1.91, 2.20]		
RTS	Injured	1.92 [1.76, 2.08]	0.15 [-0.05, 0.36]	0.23
	Uninjured	2.08 [1.91, 2.24]		
12wks	Injured	2.14 [1.95, 2.33]	-0.11 [-0.41, 0.18]	0.84
	Uninjured	2.03 [1.84, 2.22]		

^a^Between limb difference calculated as $Uninjured-Injured$

## Discussion

This prospective, observational, cross-sectional study investigated between-limb differences in SWS following HSI at TOI, RTS, and 12wks after RTS. We observed differences in SWS between the injury location in the involved limb and the matched location on the uninjured limb at TOI but not at RTS and 12wks.

Lower SWS were observed in the injured limb compared to the uninjured limb at TOI. The quantification of SWS is used to characterize the material properties of muscle, which are dictated by its hierarchical structure resulting in anisotropic behavior. For example, the propagation of ultrasound shear waves is higher in the longitudinal view compared to the transverse view in skeletal muscle due to the waves traveling preferentially along the cytoskeletal structures [21, 37, 38]. Muscle strain injury often involves structural disruption of the perimysium, which is characterized as the classic feathery pattern of edema on MRI or as the disruption of the normal echotexture in ultrasound B-mode images [12, 39]. The disruption to the perimysium significantly alters the material properties of muscle and contributes to the unconstrained dispersion of edema. Together these factors—the loss of cytoskeletal structure and relative increase in isotropy at the injury site—may explain the reduced SWS observed in the injured limb compared to the uninjured limb at the TOI.

Making comparisons of our findings to the literature is somewhat difficult as few studies have investigated the effects of ultrasound elastography or SWS following acute muscle injury. One study investigated changes in shear modulus following a sustained submaximal contraction until failure of the hamstring muscles after HSI, but the athletes studied had fully recovered prior to the investigation [40]. Two studies investigated the change in elastic modulus derived from ultrasound shear wave elastography in a contusion injury in animal models [41, 42]. In both studies, the SWS increased following injury and remained elevated throughout the study duration (21–28 days). However, an HSI with evidence of both structural disruption and edema is not the same type of injury as the contusion injury induced in these animal studies. It is possible that inflammation and edema without major structural disruption of the cytoskeleton may contribute to increased intramuscular pressures resulting in higher elastic moduli in the injured limb compared to the contralateral limb [43].

Only one study has investigated shear wave elastography changes following acute muscle injury [44]. The elastic modulus was measured throughout the healing progression and was lower in the injured limb compared to the uninjured limb at 4 weeks [44]. However, the elastic modulus was not different between limbs at 8 or 12 weeks post-injury. Our findings of acutely reduced SWS in the injured muscle are consistent with these previous findings [44]. However, our findings at RTS (median time: 29 days after injury) deviate slightly from those observed in the gastrocnemius muscle at 4 weeks. This could be due to a wider range for the clinical determination of RTS (~ 19–49 days) and the subsequent delay in SWS acquisition in our study compared to the pre-determined 4- and 8-week times in the previous investigation. Another possible explanation is that our participants were young, elite athletes compared to and older population (median (range) age: 41 (26–68) years). Recovery is longer in older individuals [45], which may explain the persistent between-limb SWS difference observed previously [44]. Consistent with our observations, a recent study found no between-limb differences in muscle shear modulus (measured after the athletes had returned to sport) in professional rugby players [46]. Though the cross-sectional design of our study does not allow for longitudinal investigation of the healing process and the corresponding changes in between-limb SWS, we speculate that the muscle structure, specifically deposition of collagen and regeneration of the extracellular matrix and connective tissue, may resolve by RTS (~ 7–27 days), resulting in no between-limb differences in SWS at RTS and 12wks [47, 48]. Taken together, our findings suggest HSI with structural disruption can be detected by lower SWS in the injured limb compared to the uninjured limb, suggesting that injury site SWS may complement traditional ultrasound or MRI for assessing injury severity. Future studies will aim to address the time course of changes in SWS following HSI and throughout rehabilitation.

This study was not without limitations. The sample size for each study time point was small, particularly for 12wks. Additionally, the time for athletes to RTS ranged from 19 to 49 days, which may decrease differences in SWS between limbs at this time point. However, we chose to anchor the study to when athletes RTS rather than a set time interval after injury (e.g., 3 weeks) in alignment with the primary aims of the larger study and to characterize muscle properties at RTS. Previous findings have noted residual edema observed in MRI at RTS [49], but it was unclear if between-limb differences in SWS would persist at this same time interval. Finally, only injuries that had evidence of edema (MRI-positive findings) were included in this study. It is unknown if the trend of lower injured limb SWS in MRI-negative injuries would be consistent with the findings here or reflect increased SWS similar to that in contusion injuries [41, 42].

## Conclusion

Ultrasound and MRI are often used to corroborate HSI diagnosis, yet typical measures of injury identification neglect inherent material properties of the muscle. Ultrasound shear wave elastography can non-invasively measure tissue elasticity in injury locations of the muscle. The SWS in the injured limb of collegiate athletes after HSI was lower compared to the uninjured limb at TOI, but not at RTS and 12 weeks after RTS. Future work will aim to provide insight as to if monitoring SWS can serve as a complementary method to conventional ultrasound or MRI for assessing initial injury, its relationship to severity, and as a measure of return to sport readiness.

## Data Availability

The images analyzed in the current study are not publicly available due to IRB restrictions. Data are available from the corresponding author upon reasonable request.

## Abbreviations

12wks: 12 Weeks after return to sport
HSI: Hamstring strain injury
MRI: Magnetic resonance imaging
RTS: Return to sport
SWS: Shear wave speed
TOI: Time of injury

## References

[1] Ekstrand J, Waldén M, Hägglund M (2016). Hamstring injuries have increased by 4% annually in men’s professional football, since 2001: a 13-year longitudinal analysis of the UEFA elite Club injury study. Br J Sport Med.

[2] Ekstrand J, Bengtsson H, Waldén M, Davison M, Khan KM, Hägglund M (2022). Hamstring injury rates have increased during recent seasons and now constitute 24% of all injuries in men’s professional football: the UEFA elite club injury study from 2001/02 to 2021/22. Br J Sports Med.

[3] Brooks JHM, Fuller CW, Kemp SPT, Reddin DB (2006). Incidence, risk, and prevention of hamstring muscle injuries in professional rugby union. Am J Sports Med.

[4] Pollock N, Kelly S, Lee J (2021). A 4-year study of hamstring injury outcomes in elite track and field using the British Athletics rehabilitation approach. Br J Sports Med.

[5] Pollock N, James SLJ, Lee JC, Chakraverty R (2014). British athletics muscle injury classification: a new grading system. Br J Sports Med.

[6] Patel A, Chakraverty J, Pollock N, Chakraverty R, Suokas AK, James SL (2015). British athletics muscle injury classification: a reliability study for a new grading system. Clin Radiol.

[7] Valle X, Alentorn-Geli E, Tol JL (2017). Muscle injuries in sports: a new evidence-informed and expert consensus-based classification with clinical application. Sport Med.

[8] Mueller-Wohlfahrt H-W, Haensel L, Mithoefer K (2013). Terminology and classification of muscle injuries in sport: the Munich consensus statement. Br J Sports Med.

[9] Douis H, Gillett M, James SLJ (2011). Imaging in the diagnosis, prognostication, and management of lower limb muscle injury. Semin Musculoskelet Radiol.

[10] Reurink G, Brilman EG, de Vos R-J (2015). Magnetic resonance imaging in acute hamstring injury: can we provide a return to play prognosis?. Sport Med.

[11] van Heumen M, Tol JL, de Vos R-J (2017). The prognostic value of MRI in determining reinjury risk following acute hamstring injury: a systematic review. Br J Sports Med.

[12] Petersen J, Thorborg K, Nielsen MB (2014). The diagnostic and prognostic value of ultrasonography in soccer players with acute hamstring injuries. Am J Sports Med.

[13] Moen MH, Reurink G, Weir A, Tol JL, Maas M, Goudswaard GJ (2014). Predicting return to play after hamstring injuries. Br J Sports Med.

[14] Wangensteen A, Almusa E, Boukarroum S (2015). MRI does not add value over and above patient history and clinical examination in predicting time to return to sport after acute hamstring injuries: a prospective cohort of 180 male athletes. Br J Sports Med.

[15] Creze M, Nordez A, Soubeyrand M, Rocher L, Maître X, Bellin MF (2018). Shear wave sonoelastography of skeletal muscle: basic principles, biomechanical concepts, clinical applications, and future perspectives. Skeletal Radiol.

[16] Roberts TJ (2016). Contribution of elastic tissues to the mechanics and energetics of muscle function during movement. J Exp Biol.

[17] Ličen U, Kozinc Ž (2022). Using Shear-Wave Elastography to Assess Exercise-Induced Muscle Damage: A Review. Sensors (Basel).

[18] Riemann BL, DeMont RG, Ryu K, Lephart SM (2001). The effects of sex, joint angle, and the gastrocnemius muscle on passive ankle joint complex stiffness. J Athl Train.

[19] Lacourpaille L, Nordez A, Hug F, Couturier A, Dibie C, Guilhem G (2014). Time-course effect of exercise-induced muscle damage on localized muscle mechanical properties assessed using elastography. Acta Physiol.

[20] Lieber RL, Fridén J (1993). Muscle damage is not a function of muscle force but active muscle strain. J Appl Physiol (1985).

[21] Taljanovic MS, Gimber LH, Becker GW (2017). Shear-wave elastography: basic physics and musculoskeletal applications. Radiographics.

[22] Mendes B, Firmino T, Oliveira R (2018). Hamstring stiffness pattern during contraction in healthy individuals: analysis by ultrasound-based shear wave elastography. Eur J Appl Physiol.

[23] Evangelidis PE, Shan X, Otsuka S, Yang C, Yamagishi T, Kawakami Y (2021). Hamstrings load bearing in different contraction types and intensities: a shear-wave and B-mode ultrasonographic study. PLoS One.

[24] Ando R, Suzuki Y (2019). Positive relationship between passive muscle stiffness and rapid force production. Hum Mov Sci.

[25] Wu CH, Chiu YH, Chang KV, Wu WT, Özçakar L (2022). Ultrasound elastography for the evaluation of plantar fasciitis: a systematic review and meta-analysis. Eur J Radiol.

[26] Dirrichs T, Quack V, Gatz M, Tingart M, Kuhl CK, Schrading S (2016). Shear wave elastography (SWE) for the evaluation of patients with tendinopathies. Acad Radiol.

[27] Dirrichs T, Quack V, Gatz M (2018). Shear wave elastography (SWE) for monitoring of treatment of tendinopathies: a double-blinded, longitudinal clinical study. Acad Radiol.

[28] Crawford SK, Wille CM, Stiffler-Joachim MR, Lee KS, Bashford GR, Heiderscheit BC (2021). Spatial frequency analysis detects altered tissue organization following hamstring strain injury at time of injury but not return to sport. BMC Med Imaging.

[29] Wille CM, Stiffler-Joachim MR, Kliethermes SA, Sanfilippo JL, Tanaka CS, Heiderscheit BC (2022). Preseason eccentric strength is not associated with hamstring strain injury: a prospective study in collegiate athletes. Med Sci Sports Exerc.

[30] Wangensteen A, Bahr R, Van Linschoten R (2017). MRI appearance does not change in the first 7 days after acute hamstring injury—a prospective study. Br J Sports Med.

[31] Heiderscheit BC, Sherry MA, Silder A, Chumanov ES, Thelen DG (2010). Hamstring strain injuries: recommendations for diagnosis, rehabilitation, and injury prevention. J Orthop Sports Phys Ther.

[32] Hickey JT, Opar DA, Weiss LJ, Heiderscheit BC (2022). Hamstring strain injury rehabilitation. J Athl Train.

[33] Le Sant G, Ates F, Brasseur JL, Nordez A (2015). Elastography study of hamstring behaviors during passive stretching. PLoS One.

[34] R Core Team (2018) R: A language and environment for statistical computing. R Foundation for Statistical Computing. Vienna, Austria. Available online at https://www.R-project.org/.

[35] Wilkinson RD, Mazzo MR, Feeney DF (2023). Rethinking the Statistical Analysis of Neuromechanical Data. Exerc Sport Sci Rev.

[36] Crawford SK, Lee KS, Bashford GR, Heiderscheit BC (2021). Spatial-frequency analysis of the anatomical differences in hamstring muscles. Ultrason Imaging.

[37] Lee HY, Lee JH, Shin JH (2017). Shear wave elastography using ultrasound: effects of anisotropy and stretch stress on a tissue phantom and in vivo reactive lymph nodes in the neck. Ultrasonography.

[38] Aubry S, Risson JR, Kastler A (2013). Biomechanical properties of the calcaneal tendon in vivo assessed by transient shear wave elastography. Skeletal Radiol.

[39] Woodhouse JB, McNally EG (2011). Ultrasound of skeletal muscle injury: an update. Semin Ultrasound CT MR.

[40] Freitas SR, Mendes B, Firmino T (2022). Semitendinosus and biceps femoris long head active stiffness response until failure in professional footballers with vs. without previous hamstring injury. Eur J Sport Sci.

[41] Zhou X, Wang C, Qiu S, Mao L, Chen F, Chen S (2018). Non-invasive assessment of changes in muscle injury by ultrasound shear wave elastography: an experimental study in contusion model. Ultrasound Med Biol.

[42] Xin Y, Liu F, Li D, Zhu J (2022). Viscoelasticity assessment for in vivo quantification of muscle contusion injury in rats using shear wave elastography. Ultrasound Med Biol.

[43] Liu J, Liao Z, Wang J, Xiang H, Zhu X, Che X, Tang Y, Xie J, Mao C, Zhao H, Xiong Y (2022). Research on skeletal muscle impact injury using a new rat model from a bioimpact machine. Front Bioeng Biotechnol.

[44] Yoshida K, Itoigawa Y, Maruyama Y, Kaneko K (2019). Healing process of gastrocnemius muscle injury on ultrasonography using B-mode imaging, power Doppler imaging, and shear wave elastography. J Ultrasound Med.

[45] Baker BA (2017). An old problem: aging and skeletal-muscle-strain injury. J Sport Rehabil.

[46] Kawai T, Takahashi M, Takamoto K, Bito I (2021). Hamstring strains in professional rugby players result in increased fascial stiffness without muscle quality changes as assessed using shear wave elastography. J Bodyw Mov Ther.

[47] Mackey AL, Kjaer M (2017). Connective tissue regeneration in skeletal muscle after eccentric contraction-induced injury. J Appl Physiol (1985).

[48] Hyldahl RD, Nelson B, Xin L (2015). Extracellular matrix remodeling and its contribution to protective adaptation following lengthening contractions in human muscle. FASEB J.

[49] Sanfilippo JL, Silder A, Sherry MA, Tuite MJ, Heiderscheit BC (2013). Hamstring strength and morphology progression after return to sport from injury. Med Sci Sport Exerc.

